# Supplementary material for: Patterns of Intron Gain and Loss in Fungi
Source: PLoS Biol. 2004 Nov 30;2(12):e422. doi: 10.1371/journal.pbio.0020422 (PMC532390; doi:10.1371/journal.pbio.0020422)
Supplement: Table S1 — Also available at http://genes.mit.edu/NielsenEtAl/. (4.3 MB ZIP). [file pbio.0020422.st001.zip › NielsenEtAl/html/409.html]

AN3005.1.NCU09711.1.MG03859.1.FG06052.1


```
 CLUSTAL W (1.82) Multiple Sequence Alignments - Introns Inserted


Sequence 1: NCU09711.1	822 aa
Sequence 2: MG03859.1	849 aa
Sequence 3: FG06052.1	813 aa
Sequence 4: AN3005.1	796 aa
Alignment Length: 858 aa
Number Identitical Residues: 405 aa
Alignment Score (without introns) 20196


MG03859.1 	MARQQPA1KIKKMFLVRNA1AKLIFGSGS-QESLIELPQGQLYLVRPLSPKGYSELIFKD
NCU09711.1	-------~----MFVLRGA1AKLLFGSGNNQETLVELPQGQLYLVRPLSPKGYSELLFKD
FG06052.1 	-------~----MFALRGI1GKLIFGSSA-QETLIELPQGQLYLVRPLSPKGYSELIFRD
AN3005.1  	-------~----MFMLRNV1SKFIFGDNS-KESIIDIPQGQLYLVRPLSPKGYSELIFKD
          	            ** :*.  .*::**..  :*:::::*******************:*:*

MG03859.1 	AAASIRRTAQDFQYQLVVQRFYEEGEAELLNE--GDEEETAEDALSSERDEKTFLLDEAL
NCU09711.1	ASARIRRTAQDFQYQLVVQRVYEEGEAELLAEEEGEDADIDADILAAERDEKTFLLDEAL
FG06052.1 	SAIRIRRTNQEFQYQLVVQRVFEEGEAELLAEEEGEDAEIDA--LASEKDEKTFLLDEAL
AN3005.1  	AAASIRRTGQEFQYQLVIQRAYEEGEEELAEDESGEGLSDSLD-----KDEKAFLLDQNL
          	::  **** *:******:** :**** **  :..*:  .   .     :***:****: *

MG03859.1 	HLRVENREGSSERVICWRDLSGDAGDVYEFVCDSSAATSAARVDDFVRIARQCQYERKYR
NCU09711.1	HLRVEQREGG-DHVLAWRDLSGDVGDVYEFVCDT--AISSSLVDQFQLAAKQCQYERKYR
FG06052.1 	CFRFEIRETG-DKVLAWKDLSGDTGDLFQFVCDS--TVTSAQVQQFVRVAQECQYERKYR
AN3005.1  	HFRSEVREGG-SKILAWRDLSGDAGDLFEFICDP--SVPSDKVSTFELAACQCQYERKYR
          	 :* * ** . .:::.*:*****.**:::*:**.  : .:  *. *   * :********

MG03859.1 	KPHTTASEADLESFD-FDEQLIPPASPIHSPS--ISRTLEANESMFSTITNTPNKAVKRA
NCU09711.1	KPNTLATEEDLQQFEFFDEPPIPQASPIHSPAL-LTRSIDSIDAMP---LHSSNTSVKRE
FG06052.1 	KSNSTATEADLQQFEFEEEDPIPPASPLHSPT--LARSIESVDDMLS--KNAANSAAKRA
AN3005.1  	RSAQKATQEELLEFSFDEERPIPSASPVASPTKTRAHSVASADSTMV--RDAEYTRSKKQ
          	:.   *:: :* .*.  :*  ** ***: **: : :::: : :       .:  .  *: 

MG03859.1 	PKPEPEPELESEPEVETAPLEAEVTPTKASKGNDREIGPPPAAAPPEATEILAAELGELH
NCU09711.1	---------NAATGSSTPAKSTPVKSTPAKK--ERNYSPPKATVPPEAREVMAAEKGELH
FG06052.1 	P--------EVIPEVVP---EQEIEPQVLTG--------PDANNPPEAIEIYAAVTAELH
AN3005.1  	---------LAAPALGD-------EPTVAPP----------SAAQPEAREVLTKERAELH
          	            .            .               :   *** *: :   .***

MG03859.1 	FFDFPSGAFVLQASSVTATVSEVGKWEYWLQISSSEKDFVGLPVIADMNPVFNFEFLSFI
NCU09711.1	FYDFPSGSFVLQADSVTATVSEIGNWKYWLQVSTPDREWLGIPISSEINPVFNPEYLSFI
FG06052.1 	VFDPQPGHFALVDDSIIAQVSEVGKWEYWLQIESGDKAYLGTPVVADFNPVFDFEYLSFV
AN3005.1  	LFDFQSGTFVMQDPDVTATVTEVGDWQYWLQISGKDKDWLGQAVVADINPVFNFEYLSFI
          	.:*  .* *.:   .: * *:*:*.*:****:.  :: ::* .: :::****: *:***:

MG03859.1 	FNHFSDHG-AVSWLVRFKDTETLERFQEGLMRALWEQLNEIKWEKIKEKERDYVTEAFAD
NCU09711.1	FNQFTDDGSAYSWLLRFKDQPTLDRFQEGLTSALWEQQNEMKWGKIKETEQNYVLDAFND
FG06052.1 	FNHFSSDGTARSWLLRFKDQSTLEKFQEAIMQAIWERLNETKWQKMQDKEREYVLDAFGD
AN3005.1  	FNHYDGIGSAYSWLLRFKDQETEERFQQGLLQALWEQLNETKWTKVKDNDKDYVLDAFQD
          	**:: . *:* ***:****  * ::**:.:  *:**: ** ** *:::.:::** :** *

MG03859.1 	LAMEDSPEAQLLEEEEEEEEEEEEEDTDDGARSEHYDSDEEDDDVETGTKGGAKNKALAV
NCU09711.1	LVLEDQP------VEEEEEEEEFEDAEDDGPQSEHYDTDEEEDDVEYKPKDTDTNKSIAV
FG06052.1 	LTMEDAPET----EEEEEEEEEEEELEDDRARDEEYDSDEENDSTFPKNADGEVNSQLAV
AN3005.1  	LTMEDADAAD---QEEEEEEEEEEEEANDGQRSEHYDSDEEQDDVVTRDEDGNINSQLAV
          	*.:**   :.    ******** *:  :*  :.*.**:***:*..     .   *. :**

MG03859.1 	GYKHDRSFVVRGSQIGVFKHTANNKLEFSTNISKVETPKGGLFSPKKVMLHNEDRNMILQ
NCU09711.1	GYKHDRSFVVRGSKIGVFKHTPSNHLEFSTNISKVETPKGELFSPTKVMLHNEDRNLVLQ
FG06052.1 	GYKHDRSFVVRGSKIGVFKHTPNNHLEFSTNISKVTTPSGKLMNPKKVMLHSEDRDLILQ
AN3005.1  	GYKHDRSFVVRGSKIGVFKHTPDNNLEFSTNISKVETPKGKLFSPKKVMLHAEDSNLILQ
          	*************:*******..*:********** **.* *:.*.***** ** :::**

MG03859.1 	NDADPNKLYRMDLEYGKVVDEWNVHEDIPVVTFAPEN~KFAQMTHEPTFLGISRNALYRI
NCU09711.1	KDGDPNKLYRMDLEYGKVVDEWKVHDDIPVVTFAPEN~KFAQMTHEPTFLGISRNALYRI
FG06052.1 	NEIDPNKLYRMDLEYGKVVDEWHVHDDIPVVTFAPEN~KFAQMTSEQTFLGVSNNALYRV
AN3005.1  	NGDDPKSLYRMDLEYGKIIDEWKIHDDIPVQTFAPES0KFSQMTSAQPFVAASHNALFRV
          	:  **:.**********::***::*:**** *****. **:***   .*:. *.***:*:

MG03859.1 	DPRLSGTKLVDSQLKQYVSKNDFSSVATTEKGHIAVASNKGDIRLFDRLGINAKTHIPAL
NCU09711.1	DPRVSGTKLVDSQLKQYASKNDFSALATTEKGYIAVASNKGDIRLFDRLGINAKTHIPAL
FG06052.1 	DPRLSGNKLVDADMKQYASKNDFSALATTEKGYIAVASNKGDIRLFDRLGIRAKTQLPAL
AN3005.1  	DPRVAGNKLVDAELKQYASKNDFSALATTEKGYLAVASNKGDIRMFDRLGINAKTHIPAL
          	***::*.****:::***.******::******::**********:******.***::***

MG03859.1 	GEPILGLDVSADGRWVLGTCRTYLLLIDALQKDGKNEGKLGFEKSFAADSKPQPRRLALT
NCU09711.1	GEPILGLDVSADGRWVLATCRNYLLLVDALQKGGKNEGKLGFEKSFAADSKPQPRRLALT
FG06052.1 	GDPISGIDVSADGRWILGTTKNYILLVDAQQKSGKNEGKLGFEKAFAATDKPHPRRLALT
AN3005.1  	GEAIIGLDVSADGRWVLATCRTYLLLIDALQKDGKNEGKLGFERSFAKDSKPQPRRLGLT
          	*:.* *:********:*.* :.*:**:** **.**********::**  .**:****.**

MG03859.1 	PEHVAQFAYETGKGVSFTPARFNTGSGQEETQIITATGPYVVEWSLRKVVAGNKAAYKIK
NCU09711.1	PEHVAQFTHETGKPVNFTPAKFNTGTGTEETSIITATGPYIIEWNLRKVLTGRKTPYLIK
FG06052.1 	PEHVAQFYHETGKPVDFTPAKFNTGEGAEETSIITATGPYIIEWNLKRILRGMKASYKIK
AN3005.1  	PAHVAQFQHETKKPISFTPARFNTGVDTEETSIVTATGPFIVTWSLKKVVAGRKDPYTIK
          	* ***** :** * :.****:**** . ***.*:*****::: *.*:::: * * .* **

MG03859.1 	RYSDEVMADDFKFGSDKNIIMALPHEVNMVDNKTLKKPTRQSIAGNALLGSG-RRSSARI
NCU09711.1	RYTDDVKADDFKFGTDKNVIVALPHEVNMVDHTRLKKPTRESIAGDFLDAAR-RGSSNRV
FG06052.1 	RYEEEVKADDFKFGSDKNVIVALPNEVNMVAKQSLKKPTRESIVGNIRLGGGSRRDSGRI
AN3005.1  	RYSEDVMADNFRFGSDKNVIVALPNEVNMVARKALQRPTRESIAGPVTPSRR----ATRW
          	** ::* **:*:**:***:*:***:***** .  *::***:**.*    .      : * 

MG03859.1 	G-TRDSGRYKLGKDDVVDSPY
NCU09711.1	GGPRTPAAQRLRREDIVNSPY
FG06052.1 	G-NQESGRYKLGRDDIVNAPY
AN3005.1  	G-------SRLGRDDIVNSPY
          	*        :* ::*:*::**
```
